# Combinatorial gene editing in mammalian cells using ssODNs and TALENs

**DOI:** 10.1038/srep03791

**Published:** 2014-01-21

**Authors:** Bryan Strouse, Pawel Bialk, Rohina A. Niamat, Natalia Rivera-Torres, Eric B. Kmiec

**Affiliations:** 1 Department of Chemistry, Delaware State University, 1200 N. DuPont Highway Dover, DE 19901

## Abstract

The regulation of gene editing is being elucidated in mammalian cells and its potential as well as its limitations are becoming evident. ssODNs carry out gene editing by annealing to their complimentary sequence at the target site and acting as primers for replication fork extension. To effect a genetic change, a large amount of ssODN molecules must be introduced into cells and as such induce a Reduced Proliferation Phenotype (RPP), a phenomenon in which corrected cells do not proliferate. To overcome this limitation, we have used TAL-Effector Nucleases (TALENs) to increase the frequency, while reducing the amount of ssODN required to direct gene correction. This strategy resolves the problem and averts the serious effects of RPP. The efficiency of gene editing can be increased significantly if cells are targeted while they progress through S phase. Our studies define new reaction parameters that will help guide experimental strategies of gene editing.

The correction of a single base mutation within the context of the human chromosome can be accomplished through the use of single-stranded oligonucleotide (ssODNs)^1^,^2^,^3^. The frequency with which these events occur is highly dependent on the introduction of a large amount of ssODN into the target cell, a type of mass action effect^4^,^5^. The frequency of the process, known as gene editing, can be enhanced by the pre-treatment with reagents or drugs that induce double strand (ds) DNA breakage^6^,^7^,^8^. Ds breakage activates the DNA damage response pathway and, in conjunction with the ends of the ssODNs, cause replication fork delay and retardation of cell cycle progression^9^,^10^. Stalled or slowed replication forks actually provide a more amenable target for ssODN by expanding the window of time that the ssODNs align in homologous register and initiate the gene editing reaction^11^,^12^,^13^. The mechanism of action and its regulation^2^ have now been partly elucidated, and as a result we know there are some adverse effects to the cell as a function of gene editing. In some cases, a genotoxicity has been reported in response to the standard ssODN vector composition which incorporates phosphorothioate linkages to prevent nuclease digestion and sustain half-life in the cell^14^,^15^,^16^. Additionally, the abundance of single-stranded DNA ends, at levels required to direct gene editing in human cells, induces a Reduced Proliferation Phenotype (RPP)^17^,^18^,^19^ in which corrected cells proliferate at a much slower rate than their unedited counterparts. Thus, over time, the level of corrected cells in a population becomes reduced by simple dilution and outgrowth of the uncorrected population.

Since ds DNA breaks enhance the frequency of gene editing, we sought a complimentary technology that would elevate ssODN-directed gene editing without leading to RPP. Transcription Activator-Like Effector Nucleases (TALENs) are chimeric enzymes that can be designed to make a unique cut in the genome after exogenous introduction of their expression vectors into human cells^20^,^21^. Since cut sites are unique, this low level of cleavage may not activate the DNA damage response pathway to the same degree as the broader spectrum DNA cleavage reagents (drugs), thereby sidestepping the collateral effect of RPP. While ssODN-directed gene editing can lead to inheritable nucleotide changes, the level of editing simply needs to be elevated for practical use in gene therapy or genomics. Thus, the main goals of this study were to (i), determine the potential of gene editing directed by both ssODNs and TALENs; (ii), establish some of the parameters and limitations of that reaction; and (iii), define conditions to bypass Reduced Proliferation Phenotype. Our results suggest that TALENs enable a lower level of ssODNs to be used in the gene editing reaction while simultaneously increasing frequency and providing a means to avoid RPP.

## Results

**Figure 1:**
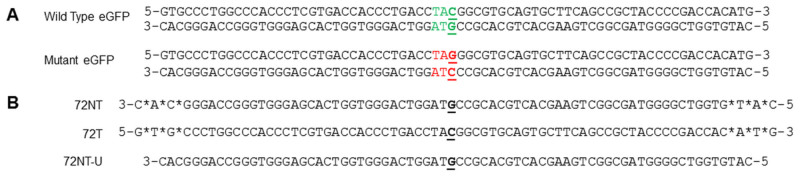
Gene editing model system and ssODNs. (A) The wild-type and mutated eGFP gene segments with the target codon located in the center of the sequences are displayed in green and red respectively. The nucleotide targeted for exchange is emphasized in bold and underlined. (B) Phosphorothioate modified, end protected (denoted with *) 72NT, a 72-mer which is used to target the non-transcribed (NT) strand and 72T, which directs exchange on the transcribed strand (T) of the mutated eGFP gene as shown. Also depicted is the sequence of the unmodified ssODN, 72NT-U, which is equivalent to the 72NT without phosphorothioate end modifications.

Gene editing activity is measured by the correction of a single base mutation in an eGFP gene integrated into HCT116 cells, the clonally expanded cell line known as HCT116-19^23^. This system has been used as the workhorse for mechanism of action and regulation studies in mammalian cells. Introduction of a specific ssODN by electroporation, lipofection or nucleofection at a level where it can direct correction of the mutant base results in the expression of a wild type eGFP transcript and the associated protein. Quantitation of gene editing is carried out by FACS which is also used to measure viability. Segments of the wild type eGFP gene sequence and its mutant analog are displayed with three ssODNs (Fig. 1); 72NT, a 72 mer that hybridizes to the non-transcribed strand and, 72T, a 72 mer that hybridizes to the transcribed strand. In addition, we present the sequence of 72NT-U, a 72 mer that hybridizes to the nontranscribed strand but does *not* contain the phosphothioate linkages among the three terminal bases. 72NT and 72T contain these linkage modifications.

**Figure 2:**
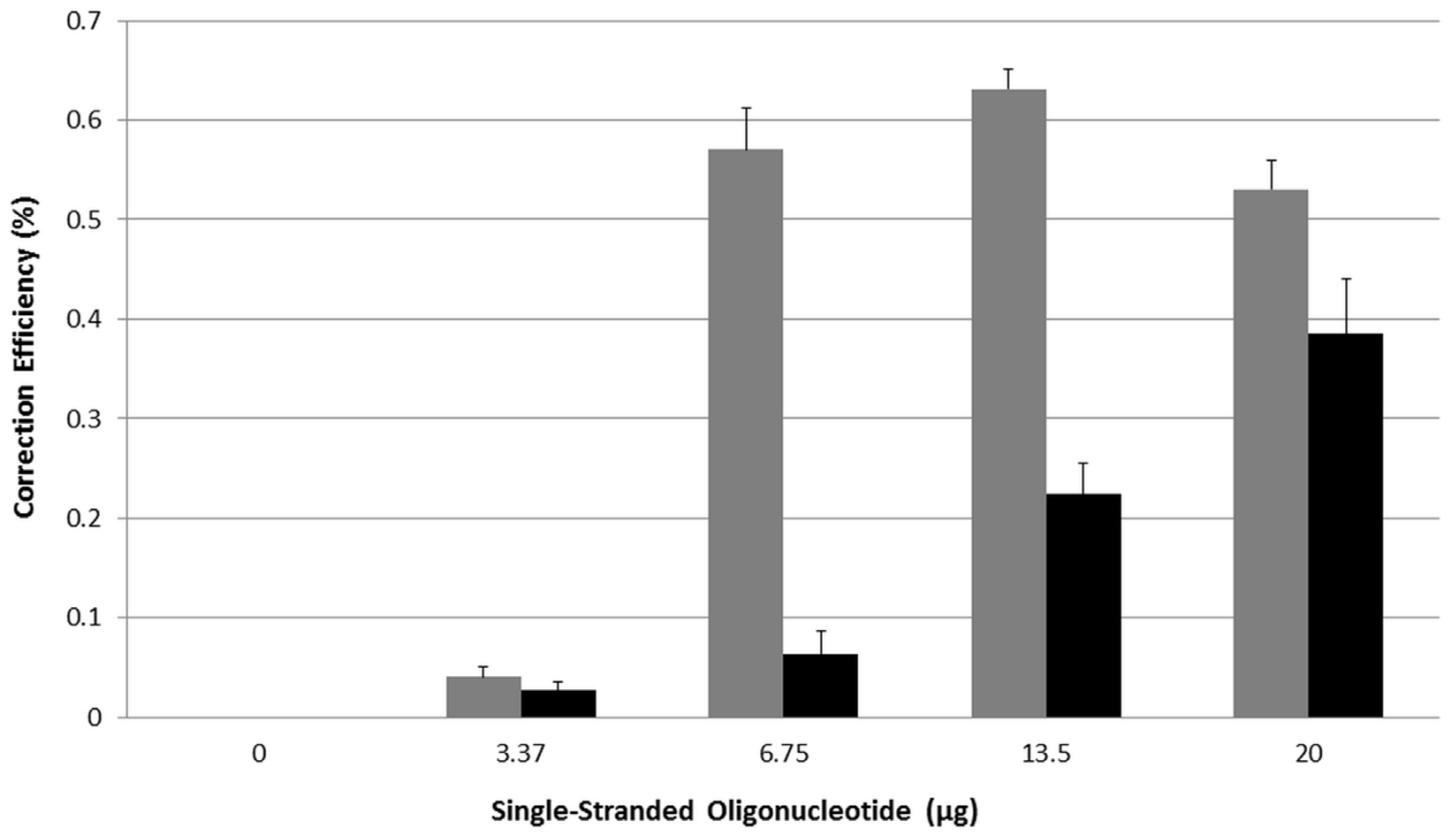
Gene editing dose curve using modified and unmodified ssODNs. Unsynchronized HCT116-19 cells were electroporated with 0, 3.75, 6.75, 13.5 and 20 ug of 72NT (light gray bars) or 72NT-U (black bars). After a 48-hour recovery period, gene editing activity was measured using a Guava EasyCyte 5 HT flow cytometer. Gene editing is displayed as correction efficiency (%), determined by the number of viable eGFP positive cells divided by the total number of viable cells in the population. Each treatment was performed in triplicate and error bars represent standard error determined by standard methods.

Figure 2 exhibits a dose curve of gene editing activity after 48 hours directed by either 72NT or 72NT-U. Since these cells are not synchronized and released, the level of eGFP^−^ gene correction is predictably low throughout. But, detectable levels of gene editing are seen at different dosages of the modified and unmodified ssODN; 72NT appears to be consistently more effective at the moderate levels while 72NT-U requires a higher dosage to produce even a modest level of correction, as reported previously^17^.

**Figure 3:**
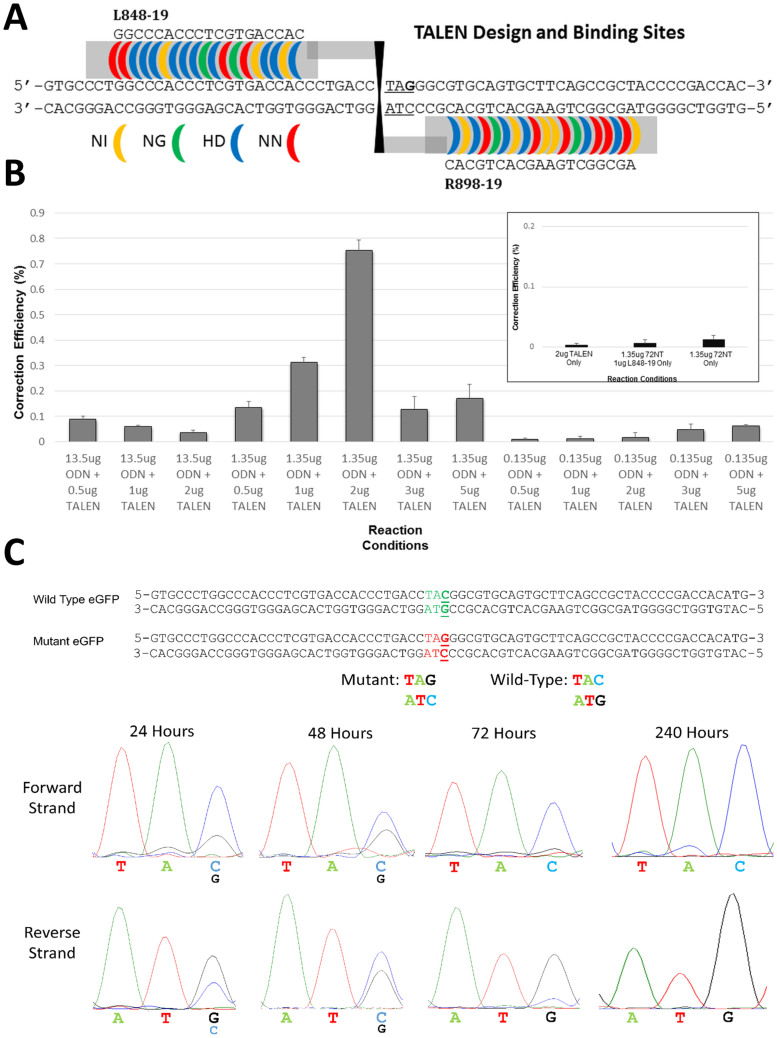
TALEN design and Gene Editing using TALENs and ssODNs. (A). The TALEN pair, designed and built using the Golden Gate method, induces a double stranded break immediately preceding the mutant codon. RVDs are shown as color coded binding blocks next to their respective base, yellow NI:A, green NG:T, blue HD:C and red NN:G. Fok1 domains are shown in black and are positioned at their predicted cut site. (B). Unsynchronized HCT116-19 cells were harvested electroporated at a concentration of 5e5 cells/100 ul with TALENs and/or 72NT ODN at the indicated amounts. TALEN amounts reflect the total TALEN plasmid added to each sample in equal portions (1 ug L848-19 and 1 ug R898-19). Left and Right TALENs must be present for a DSB to be made. Following electroporation, cells were placed in 6-well plates and allowed to recover for 48 hours. Analyses took place on a Guava EasyCyte 5 HT flow cytometer (see Materials and Methods). Correction efficiency (%) was determined by the number of viable eGFP positive cells divided by the total viable cells in the population. Each treatment was performed in triplicate and error bars represent standard error. (C). Synchronized HCT116-19 cells were electroporated under the following conditions; 2 ug TALEN and 1.35 ug 72NT at 5e5 cells/100 ul. Cells were then sorted for GFP+ at 24, 48, 72 and 240 hours post electroporation. Immediately following cell sorting, DNA was isolated and the region surrounding the target base was amplified via PCR. Samples were submitted to Genewiz (South Plainfield, NJ) for sequencing analysis.

Low levels of correction can be enhanced by simultaneous nondiscriminant ds DNA cleavage, originally induced by the addition of anticancer drugs such as camptothecin^7^. Here, we sought less broad spectrum DNA cleavage by employing Tal-Effector Nucleases (TALENs)^20^ to cut at a specific site, 5′ to the mutant base pair. The TALEN pairs used in this study were designed according to previously published guidelines^22^,^24^,^25^ for high DNA binding affinity and cutting efficiency. Construction of the TALENs followed the original Golden Gate Assembly method with a slight modification to include the Goldy TALEN backbone. Antibiotic selection and colony PCR was performed to confirm correctly constructed TALENs, and corrected clones were sent for sequence confirmation (Genewiz Inc, South Plainfield, NJ). Figure 3A represents a schematic of the TALEN used in these studies. The two arms, L848-19 and R898-19 bind to the indicated bases using the standard RVD code of NI, NG, HD and NN respectively. The spacer region of 13 nucleotides positions the FOK1 domains so that they dimerize and initiate DNA cleavage. The cut site is 5′ to the target codon, TAG. The top strand in the diagram (5′-3′) represents the non-transcribed strand to which 72NT hybridizes.

Plasmids containing TALEN expression constructs L848-19, and R898-19, were electroporated with 72NT into HCT116-19 cells under the reaction conditions presented in Figure 3B. A parallel dose curve of plasmid DNA relative to ssODN was carried out along with controls that are used routinely to validate the readout system. In parallel, 72NT levels were varied 100-fold with plasmid levels ranging from 0.5 μg to 5 μg respectively. At 1.35 μg ssODN, a clear improvement in gene editing activity is observed as a function of added TALEN dosage; at a 1.35 μg ssODN/2 μg TALEN ratio, the highest level of gene editing is seen. As the level of TALEN increases, gene editing activity decreases; and a clear optimal range is evident. Neither higher nor lower levels of ssODN, beyond 1.35 μg, direct significant levels of correction no matter what amount of TALEN expression construct is added.

These data suggest that ssODNs and a TALEN designed to cleave at the 5′ side of the mutant base in the eGFP^−^ gene carry out effective gene editing. Activity is dependent on the proper levels of ssODN and TALEN plasmids being present, which may signal a cooperative interaction among the molecules; gene editing is dependent on having both ssODNs and TALENs present in the same reaction. To confirm the specificity of gene editing at the target base, we isolated eGFP^+^ cells from various time points following electroporation of a reaction mixture containing 1.35 μg ssODN and 2 μg of TALEN plasmids by FACS. Genomic DNA was prepared and DNA sequencing was carried out across the targeted region. Figure 3C displays the results of several samples isolated under these conditions; all contain the changed DNA base at the predicted site; the specific base change G → C is shown. These data show that complete correction from TAG to TAC takes place over a 72 hour time frame and mixed peaks are seen in the 24/48 hour time period. Thus, the incorporation/replication model of gene editing as proposed by Parekh-Olmedo and Kmiec^1^ may be one of the pathways taken when the reaction is coupled by TALEN cleavage activity. Taken together, these data suggest that the ssODN/TALEN combinatorial approach leads to precise gene editing at an ssODN level that, by itself, directs undetectable levels.

Based on the data presented in Figure 3B, we established the 1.35 μg/2 μg ratio for gene editing as a foundational condition and then examined several reaction parameters surrounding it; these are presented as Supplemental Data (see Supplemental Figures Online). In this series of experiments, we changed the ssODN, using a 72-mer (72T) that is complimentary to the transcribed strand instead of 72NT. The level of gene editing is approximately half (0.3%) of the level supported by the 72NT/TALEN combination. These data align with previous observations that show gene targeting with the NT ssODN directs a higher level of gene editing than ssODNs that can hybridize to the transcribed strand. Similar results are seen when a 72 mer (NT) lacking phosphorothioate linkages (Fig. 2) is used instead of the standard 72NT; a significantly lower level of gene editing activity is observed. Mixing the 72NT/72T ssODNs at equimolar levels does not rescue the low gene editing levels seen when 72NT is used alone. A totally unrelated, scrambled 72 mer, 72NS, does not direct detectable levels of gene editing, results that align with previous observations addressing the specificity of ssODN gene editing^26^,^27^,^28^,^29^. The reaction is dependent on TALENs being expressed to achieve the significant levels of correction especially at the low levels of ssODN used in these reactions (see Supplemental Fig. S1 online).

**Figure 4:**
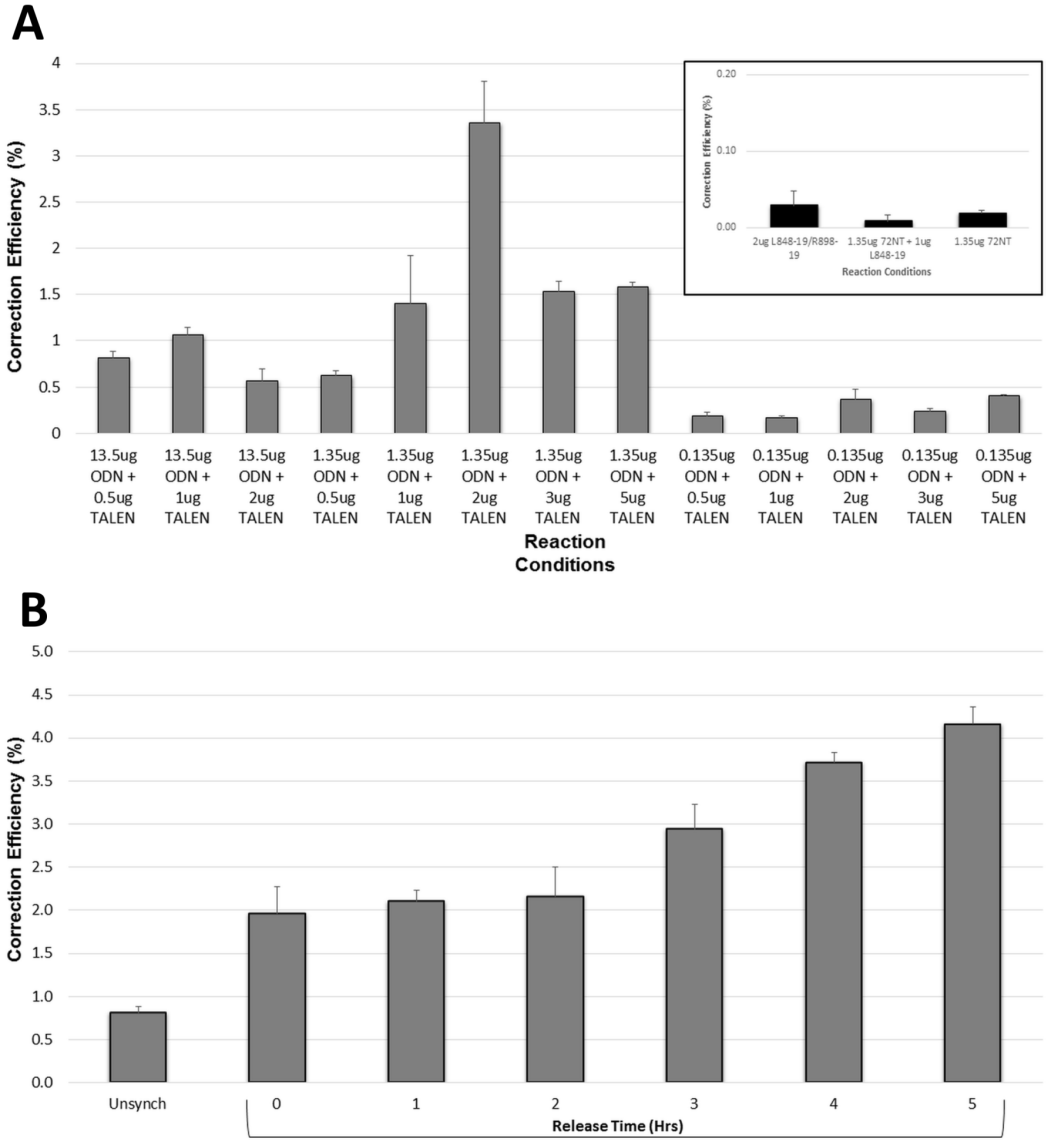
Gene editing of synchronized and released HCT116-19 cells using TALENs and ssODNs. HCT116-19 cells were seeded at 2.5e6 cells in a 100 mm dish and synchronized for 24 hours with 6 uM aphidicolin then released for 4 hours before being electroporated at a concentration of 5e5 cells/100 ul with TALENs and/or the 72NT ODN at the amount indicted. TALEN amounts reflect the total TALEN plasmid added to each sample in equal portions (1 ug L848-19 and 1 ug R898-19). Left and Right TALENs must be present for a DSB to be made. Following electroporation, cells were seeded in 6-well plates and allowed to recover for 48 hours and analyses took place on a Guava EasyCyte 5 HT flow cytometer (see Materials and Methods). Correction efficiency (%) was determined by the number of viable eGFP positive cells divided by the total viable cells in the population. Each sample set was performed in triplicate and error bars represent standard error. (B). HCT116-19 cells were synchronized at the G1/S border with 6 uM aphidicolin for 24 hours; 5e6 cells/100 ul were then electroporated with a total of 2 ug TALEN at equal levels and 1.35 ug 72NT at 0, 1, 2, 3, 4 and 5 hours respectively, after release from aphidicolin. Following a 48 hr recovery, cells were analyzed for correction efficiency (%) via flow cytometry. Correction efficiency (%) was determined by the number of viable eGFP positive cells divided by the total viable cells in the population. For comparison, a population of unsynchronized cells treated similarly is also shown.

The levels of gene editing activity can be raised if the targeted population contains a preponderance of cells in S phase^9^,^30^,^31^. In fact, a slowing of S phase progression can enhance the frequency even further. Our lab has used the synchronization and release experimental design routinely and thus we tested the impact of cell cycle manipulation on correction directed by ssODNs and TALENs. We used aphidicolin to synchronize cells at the G1-S border and then released the population for 4 hours. At that time, the 72NT-mer and the TALEN constructs were introduced by electroporation. The conditions and reagent combinations described in Figure 3B were repeated on these synchronized and released cells. As seen in Figure 4a, the patterns of gene editing activity are broadly consistent with the results obtained with unsynchronized cells. In fact, there appears to be a simple amplification of gene editing activity when synchronized/released cells are used. Again the 1.35 μg/2 μg ssODN/TALEN levels are optimal and neither increasing nor decreasing the ssODN amount positively affects the reaction. Thus, as with ssODN-directed correction, cells actively progressing through S phase provide a more amenable target for gene editing. Considering the steric hindrance that complex chromatin structures would present to both ssODNs and TALENs, metabolic processes that help unwind chromosomal architecture are likely to enhance target-site accessibility. The same levels of unprotected (unmodified) ssODN and TALEN (at 2 μg) are seen to be optimal in directing the gene editing reaction (see Supplemental Fig. S2 online).

In 2008, Engstrom and Kmiec^10^, examined gene editing in S phase in more detail. They found that the release time prior to electroporation can have an effect on the level of gene editing activity. The level maximizes at approximately 4 hours after release. We repeated that experiment using ssODNs and TALENs at the optimal levels (1.35 μg/2 μg) respectively. These reaction components were added at 0–5 hours after release from the synchronization block; a steady increase in gene editing activity is seen up until 4–5 hours post release (Fig. 4B). These results are consistent with data from Engstrom and Kmiec^10^,^32^, perhaps indicating that the ssODN/TALEN combinatorial approach is influenced by some of the same factors that have been found to impact ssODN-directed DNA targeting. Again, at 4–6 hours the cell population appears to be in mid S phase, the previously identified time point for maximal gene editing activity.

**Figure 5:**
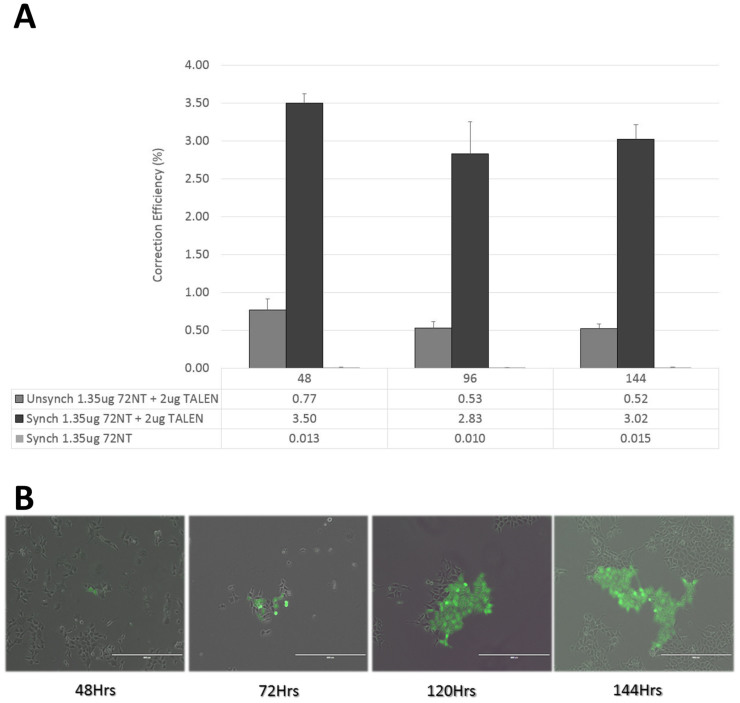
Recovery of corrected cells as a function of time. Unsynchronized (dark gray bars) and synchronized (black bars) cells were electroporated with 2 ug TALEN and 1.35 ug 72NT. Synchronized and released cells were also electroporated with 1.35 ug of 72NT only (light gray bars). Flow cytometry was used to measure correction efficiency over time at 48, 96 and 144 hours respectively, post-electroporation. Correction efficiency (%) was determined by the number of viable eGFP positive cells divided by the total number of viable cells in the population. Each treatment was performed in triplicate and error bars represent standard error. (B). Transmitted and GFP channel images of HCT116-19 cells were acquired at 48, 72, 120 and 144 hours were merged together to observe the expansion of GFP positive, corrected cells over time. Images were acquired using the EVOS FL (AMG Micro, Bothell, WA) microscope at a magnification of 10×.

**Figure 6:**
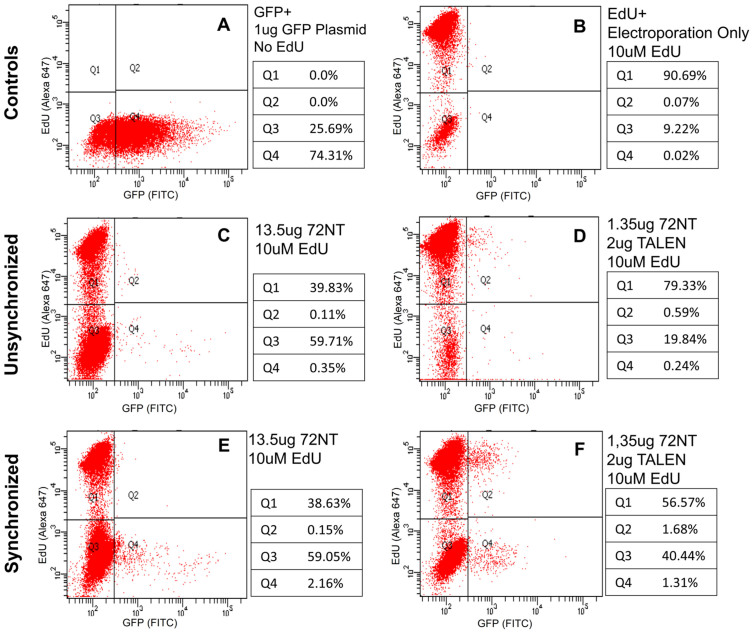
DNA replication activity of gene edited cells. Unsynchronized and synchronized cells were electroporated with either 13.5 ug 72NT or 2 ug TALEN plus 1.35 ug 72NT. Following a 48 hr recovery period, 10 uM of EdU was added to each sample and incubated for 18 hours (>one DNA replicative, division cell cycle). Immediately after EdU incubation, Click-iT EdU assay was performed to identify actively replicating cells by FACS. Quadrant 1 displays replicating but uncorrected cells; quadrant 2, replicating and corrected; quadrant 3 is nonreplicating and uncorrected; and quadrant 4 is nonreplicating and corrected. Percentages of cells in each quadrant were calculated automatically by FACSARIA II and are presented adjacent to each profile.

Recently, we reported that the level of ssODN required to direct measurable gene editing activity induces a Reduced Proliferation Phenotype (RPP). The number of ssODN ends introduced into the cell activate the DNA damage response pathway, specifically cell cycle checkpoint proteins Chk1 and Chk2^4^,^5^. Both DNA replication and cell division are stopped in the corrected cells, i.e., those cells that have received enough ssODN to undergo gene editing. This phenomenon is manifested by a gradual reduction in correction frequency over time [see^17^,^18^,^19^]. Since TALENs enable the level of ssODN to be reduced 10 fold, we expanded the time course of editing between 48 hours and 144 hours and measured activity to see if the initial level is generally maintained. Figure 5A displays the results–no reduction in correction efficiency is observed up through 144 hours in either cells that have been synchronized and released (4 hour point) or cells that are unsynchronized when targeted. The impact of the level of synchronization on ssODN/TALEN-directed gene editing is evident once again. These results stand in sharp contrast to previously published work on extended incubation times when gene editing is directed by ssODNs alone^17^. Gene editing activity directed by single-stranded ODNs alone is also presented and displays almost undetectable levels. At 1.35 μg, ssODNs do not catalyze gene editing, emphasizing the importance of the TALEN in the reaction mixture. Similar controls are seen in Figure 2 as well while testing unsynchronized cells revealed absolutely no gene conversion (data not shown, but see 17, 18). Images of the dividing and corrected cells (at various reaction times) are presented in Figure 5B, reflecting the FACS data shown in Figure 5A. Finally, we wanted to confirm that at least some of the corrected, eGFP^+^ cells were undergoing DNA replication and cell division. Previously, we utilized a Click-iT assay, using EdU to identify cells bearing active replication forks. The cells were targeted then allowed to recover for 48 hours, at which time EdU was added. FACS was carried out 18 hours later measuring gene editing (generation of eGFP^+^ cells) on the x axis and on the y axis, EdU incorporation (evidence of DNA replication activity). Thus, four quadrants representing differing combinations, are created: Q1, replication positive and non-corrected; Q2, replication positive and corrected; Q3, replication negative and non-corrected; Q4, replication negative and corrected. The six panels A–F in Figure 6 illustrate the results. Panels A and B serve as controls with A exhibiting the capacity of HCT116 cells to express eGFP from a standard plasmid expression construct and be detected by FACS (74.3%), and B, demonstrating efficient uptake of EdU by HCT116-19 cells (90.69%) during the 18 hours of incubation time. Panel C displays the low level of correction directly by 13.5 μg of 72NT alone as seen previously in Figure 2. Only 0.1% of the corrected cells score positive for active replication. In contrast and using one tenth the level of ssODN plus TALEN, panel D reveals 0.59% of cells corrected and in replicative form. Panels C and D represent experiments carried out with unsynchronized cells. Finally, panel E represents gene editing activity directed by 13.5 μg of 72NT in synchronized and released cells. While the total correction level is approximately 2.3%, only 0.15% (<7% of corrected cells) harbor active replication forks. In contrast, using one tenth the amount of 72NT (plus TALEN), a full 1.7% of the corrected cells (total 3%) display DNA replication activity. Taken together, these data demonstrate that gene editing with ssODNs alone result in the majority of corrected cells being negative for DNA replication (RPP). In contrast, the majority of cells, corrected by very modest amounts of ssODN and TALENs are capable of maintaining their capacity to replicate. Thus, the use of TALENs in combination with ssODNs, reduces the level of targeting molecule needed for the reaction, elevates the frequency and, as a result, the majority of cells avoid RPP.

## Discussion

Gene editing directed by ssODNs, takes place in at least three definable phases (1, 2). The first, initiation, involves the alignment of the ssODN in homologous register with the target site. Next, the step of correction comprises the actual nucleotide exchange and last, recovery is the phase in which the cell resumes its normal metabolic activities. The mechanism of action involves the incorporation of the ssODN into a growing replication fork^2^,^3^,^13^, which, in all likelihood, disrupts the chromatin structure, reduces steric hindrance and permits ssODN access to the target site. Yet the amount of ssODN required to direct gene editing is quite high leading to the cellular phenomenon called the Reduced Proliferation Phenotype (RPP) within which the corrected cells fail to replicate their DNA and do not divide^18^. This phenomenon is particularly apparent in the results presented in Figure 6. If one compares quadrants 1 and 2, panels C and D respectively, the dramatic reduction in replication activity as a function of the addition of 13.5 μg of ssODN to facilitate editing, can be readily observed. Here only 39.9% of the cells reveal active replication whereas in the presence of one tenth the level of ssODN, 79.9% are replication positive (panel D). The DNA damage response pathway is activated by these high numbers of single stranded DNA ends in the reaction and, as a result, cell cycle progression is slowed dramatically. Aarts et al.^3^ have suggested the gradual loss of eGFP^+^ cells is a result of semi-conservative replication in which the ssODN was incorporated into the transcribed strand. While we see RPP engaged whether a T or NT ssODN is used (or incorporated) this insightful explanation of mechanism can be tested experimentally. A major challenge in advancing gene editing toward clinical implementation is still to improve target accessibility, elevating frequency of correction.

In this paper, we demonstrate that TALENs can act (with ssODNs) to carry out gene editing. This combinatorial approach increases the frequency of the reaction dramatically and cells bearing that altered base proliferate normally. TALENs appear to coordinate this response in several ways. First, they can be designed to create a ds break within the target gene near the mutant nucleotide, here TA**G** in the mutant eGFP gene. Double strand DNA breakage has been shown previously to raise the frequency of gene repair many fold^6^,^7^ and thus, by the very nature of their enzymatic activity, TALENs stimulate the reaction. Second, since TALENs can cleave chromosomal DNA, the disruption of the physical barrier to target accessibility enables the alignment of the ssODN at the target site with a higher degree of efficiency. This reaction is reminiscent of the destabilization effects on chromatin conferred by DNA replication and HDAC inhibitors. Pre-treatment of targeted cells with Na-Butyrate or TSA has been shown to enhance gene editing frequencies^33^. And third, TALENs act to reduce the amount of ssODNs needed to direct the reaction and, as such, activation of the DNA damage response is averted. Thus, a more compatible environment for the re-establishment of DNA replication and ultimately cell division is created.

A number of investigators have been developing the combinatorial approach of TALEN/ssODNs for genome editing in engineered embryonic stem cells and zebrafish. Wefers *et al.* have used microinjection to deliver TALENs and ssODNs into mouse ES cells creating and correcting chocolate missense mutations in RAB38^34^. Ding *et al.* generated 15 mutant alleles in somatic and human pluripotent stem cells, the latter being effectively differentiated into a variety of metabolic cell types^35^. These workers carried out a comprehensive study demonstrating target specificity and the capacity to generate isogenic cell lines for modeling human diseases *in vitro*. Bedell *et al.* showed germline transmission of a *mloxP* site and a “custom-designed” *EcoRV* site, created by TALEN/ssODN action, in zebrafish^22^. Briggs *et al* demonstrated important parameters about TALEN positioning on the target site while measuring gene editing in an eGFP reporter model system; albeit with optimizing reaction conditions^36^. This type of evidence aligns with our current data and the notion of the universal potential for the combinatorial approach.

Our data suggest that both modified and unmodified ssODNs can be used with TALENs for gene editing, although modified ssODNs still produce the highest levels of corrected cells^23^. In this work, a single TALEN (L848-19/R898-19) was designed to cut 5′ relative to the targeted codon and while this improves the reaction significantly, other TALENs may produce even higher levels of correction. We are currently optimizing the position of TALEN cleavage as it relates to improved gene editing activity. Our current studies reveal that gene editing is enabled in both unsynchronized and synchronized released cells if both TALEN arms are present, the ssODN has complimentary sequence to the target site and an optimized ratio of ssODN/TALEN is utilized. Gene editing activity appears to be dose dependent when ssODNs and TALENs are used together; increasing the amounts of TALEN elevates the correction up to a point (Figs. 3B and 4A). But, there is an optimal level of TALEN since higher doses do not continue the upward trend. These observations may suggest that the ssODN entry into the duplex must be coordinated with TALEN cleavage and too much ds breakage is counterproductive; the corrected sequence may be cut and rendered non-functional.

The enhancement of gene editing frequency in cells that have been synchronized and released provides us with some insight into the mechanism of action of this combinatorial approach. As we proposed before^1^, ssODNs that incorporate into a growing replication fork act as a quasi-“Okazaki fragment” priming the elongation of the newly replicated strand^14^. Subsequent rounds of replication result in the evolution of gene edited cells; this still could be a general mechanism of gene editing, promoted by TALENs and ssODNs (see Figure 3B, 24/48 hour time points). But, the data presented in Figure 6 (and Fig. 5A) may suggest an alternative route as well. The process of correction could involve an ssODN mediated repair, bridging the cleavage point (created by the TALEN action) with dual correction of both strands. The activity of the TALEN may also provide an entry point for the ssODN to more easily navigate the structural hindrance of chromatin and to identify its complimentary binding sites in the DNA. For most applications such as gene knock-out, Non-Homologous-End-Joining (NHEJ) is the prevalent form of DNA re-joining. Knock-outs likely arise from the resection of DNA that accompanies the NHEJ process. But, in the case of gene editing, we seek a more precise reaction outcome, in terms of DNA integrity. Since we need to preserve reading frame, it is likely that ssODN/TALEN-directed gene repair may follow the homologous recombination pathway more often, although one may not exclude NHEJ. In fact, recent data suggest NHEJ and HR may not be mutually exclusive when ssODNs are present [see 37 and references therein]. A number of recent observations are pertinent to this view. First, gene editing is highly active in S/G2 phases, and much less active in G1 or G0^5^,^9^,^10^,^38^. Second, (as stated above) ds breakage enhances gene editing^6^,^7^, TALENs provide a tool to achieve ds breakage at a unique location. Third, evidence exists for the incorporation of the ssODN into the duplex as a part of the gene editing reaction^14^,^36^. Thus, we suggest that once the ssODN enters the duplex at the break site facilitated by TALEN action, it serves as a patch or bridge enabling the repair reaction, catalyzed by homologous recombination to take place. This model, while early in development, is consistent with our observations surrounding the mechanism of gene editing. Here, as proposed by Liu *et al*, in an elegant study, the ssODNs may actually compete for ends that…”would otherwise enter(ed) the NHEJ pathway”^37^. In addition, Morozov and Wawrousek (2008) found that inhibiting the activity of proteins involved in NHEJ actually increased the frequency of gene editing^39^. These observations are consistent with the notion that gene editing ssODNs proceeds through the process of HR when ds breaks proceed and enable ssODN annealing. Thus, we suggest that the fundamental role of the TALEN is to disrupt chromatin structure and define an entry site near the target base for the ssODN to initiate the annealing process. A number of simple predictions can be made to test this hypothesis including changing the cleavage site relative to the target base (or codon), a design modification that should impact gene editing frequencies. Yang et al.^40^ defined an optimal series of cut sites in the CCR5 gene of a stem cell genome which align with our own observations (Rivera-Torres et al. in preparation) and suggest that the highest frequency of gene editing is enabled when the cleavage takes place within 25–30 base pairs relative to the target base. If the mechanism of TALEN/ssODN-directed gene editing can be elucidated even partially, we should be able to design a combinatorial approach to impact monogenic diseases in a more rational way. Experiments aimed at exploring these facets of gene editing are now underway in our laboratory.

## Methods

### Cell line and culture conditions

HCT116 cells were acquired from ATCC (American Type Cell Culture, Manassas, VA). HCT116-19 was created by integrating a pEGFP-N3 vector (Clontech, Palo Alto, CA) containing a mutated eGFP gene. The mutated eGFP gene has a nonsense mutation at position +67 resulting in a nonfunctional eGFP protein. For these experiments, HCT116 (-19) cells were cultured in McCoy's 5A Modified medium (Thermo Scientific, Pittsburgh, PA) supplemented with 10% fetal bovine serum, 2 mM L-Glutamine, and 1% Penicillin/Streptomycin. Cells were maintained at 37°C and 5% CO_2_. Custom designed oligonucleotides, 72NT, 72T and 72NT-U were synthesized from IDT (Integrated DNA Technologies, Coralville, IA).

### TALEN design and construction

TALENs of 19 RVDs each were designed to flank the target site of the integrated mutant eGFP gene to bind to the following sequences: L848-19 5′GGCCCACCCTCGTGACCAC and R898-19 5′ AGCGGCTGAAGCACTGCAC. TALEN Construction was done via the Golden Gate Assembly method originally developed by Cermak et al.^20^ and purchased through Addgene (Addgene, Cambridge, MA). The final step of the assembly protocol was modified to include the mammalian expression vector pc-GoldyTALEN, which has optimized for expression and cutting efficiency in mammalian systems^22^. Following construction, colony PCR and DNA sequencing by Genewiz Incorporated (South Plainfield, NJ) was performed to confirm correct TALEN constructs.

### Transfection of HCT116-19 cells and experimental approach

For experiments utilizing synchronized cells, HCT116-19 cells were seeded at 3.0 × 10^6^ cells in a 100 mm dish and synchronized with 6 μM aphidicolin for 24 hours prior to targeting. Cells were released for 4 hours (or indicated time) prior to trypsinization and transfection by washing with PBS (−/−) and adding complete growth media. Synchronized and unsynchronized HCT116-19 cells were transfected at a concentration of 5 × 10^5^ cells/100 ul in 4 mm gap cuvette (BioExpress, Kaysville, UT). Single-stranded oligonucleotides and/or TALEN plasmid constructs were electroporated (250 V, LV, 13 ms pulse length, 2 pulses, 1 s interval) using a Bio-Rad Gene Pulser XCell™ Electroporation System (Bio-Rad Laboratories, Hercules, CA). Cells were then recovered in 6-well plates with complete growth media at 37°C for the indicated time prior to analysis.

### Analysis of gene edited cells

Fluorescence (eGFP) was measured by a Guava EasyCyte 5 HT Flow Cytometer (Millipore, Temecula, CA). Cells were harvested by trypsinization, washed once with 1× PBS (−/−) and resuspended in buffer (0.5% BSA, 2 mM EDTA, 2 μg/mL Propidium Iodide (PI) in PBS −/−). Propidium iodide was used to measure cell viability as such, viable cells stain negative for PI (uptake). Correction efficiency was calculated as the percentage of the total live eGFP positive cells over the total live cells in each sample. Error bars are produced from three sets of data points generated over three separate experiments using basic calculations of Standard Error.

Sequence confirmation of ssODN/TALEN edited cells was carried out by fluorescence-activated cell sorting of eGFP+ cells via the BD FACSAria II sorter - 488 nm (100 mw) (BD Biosciences, San Jose, CA). 1.35 ug 72NT and 2 ug TALEN transfected cells were sorted at 24,48,72 and 240 hours post electroporation. Immediately, DNA was isolated from each sample was using the Blood and Tissue DNeasy kit (Qiagen, Hilden, Germany). The targeted site was amplified via PCR using forward primer, 5′CTGGACGGCGACGTAAACGGC and reverse primer, 5′ ACCATGTGATCGCGCTTCTCG. PCR cleanup was performed using the QIAquick® PCR purification kit (Qiagen, Hilden, Germany) and the purified samples were sent for sequencing to Genewiz Incorporated (South Plainfield, NJ).

### HCT116-19 click-iT EdU cell proliferation assay

Gene editing reactions were carried out on HCT116-19 cells as described above. At 48-hours post transfection, EdU was added at a concentration of 10 μM to label actively replicating DNA in cells. EdU incubation was carried out for 18 hours (>one cell cycle) to ensure all cells had time to proceed through S-phase. Following EdU incubation, cells were washed 2× with PBS (−/−), harvested by trypsinization and processed using the Click-iT EdU Alexa Fluor 647 Flow Cytometry Assay Kit (Life Technologies, Carlsbad, CA). Briefly, cells were fixed for 15 minutes at room temperature with Click-iT fixative. After fixation, cells were washed with buffer, and permeabilized for 15 minutes using saponin-based permeabilization wash reagent followed by incubation with the Click-iT reaction cocktail containing the Alexa Fluor 647 azide for 30 minutes. Anti-eGFP Alexa Fluor 488 antibody was then applied for 60 minutes to identify eGFP expressed in edited cells since the natural fluorescence of eGFP is quenched by the Cu^2+^ contained in the Click-iT reaction cocktail. After incubation, the cells were resuspend in wash buffer and analyzed by flow cytometry using the BD FACSAria II sorter (BD Biosciences, San Jose, CA). EdU uptake was analyzed by the 633 excitation laser with a 670/30 emission filter (y-axis), while FITC (GFP expression) was attained through the 488 laser with a 530/30 emission filter (x-axis).

## Author Contributions

B.S. and P.B. designed, constructed and supplied the TALENs; B.S., P.B., R.N. and N.R.T. carried out the gene editing reactions; B.S., P.B. and E.B.K. designed experimental flow: E.B.K. did the primary writing of the manuscript.

## Supplementary Material

Supplementary Information Supplemental Figure S1 and S2
