# Supplementary material for: Combinatorial gene editing in mammalian cells using ssODNs and TALENs
Source: Sci Rep. 2014 Jan 21;4:3791. doi: 10.1038/srep03791 (PMC3896902; doi:10.1038/srep03791)
Supplement: Supplementary Information — Supplemental Figure S1 and S2 [file srep03791-s1.pdf]

Supplemental Information

Combinatorial gene editing in mammalian cells using ssODNs and TALENs

Bryan Strouse, Pawel Bialk, Rohina A. Niamat, Natalia Rivera-Torres and Eric B. Kmiec*

Department of Chemistry, Delaware State University, 1200 N. DuPont Highway Dover, DE 19901
(302) 857-6530 (phone); (302) 857-6539 (Fax)

Running title: Genome editing with single-stranded DNA oligonucleotides and TALENs

*corresponding author

*To whom reprint request should be addressed: Eric B. Kmiec, Ph.D, Professor and Chairman, Delaware State University, Department of Chemistry, 1200 N. DuPont Highway, Dover, Delaware 19901-2277; 302-857-6530 (phone), 302-857-6539 (Fax), ekmiec@desu.edu (Email), www.kmieclab.com

Supplemental Figure S1

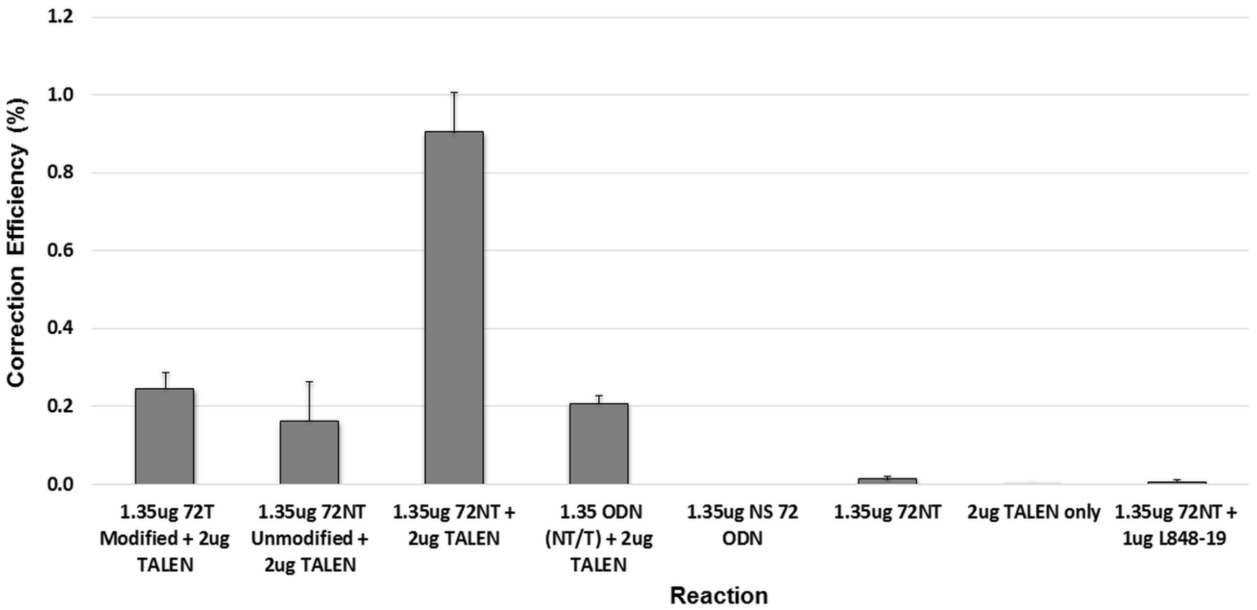

Supplemental Figure S1: Gene editing of HCT116-19 cells using TALENs and ssODNs. Unsynchronized HCT116-19 cells were harvested and electroporated at a concentration of 5e5 cells/100ul with the indicated TALEN pair and/or various ssODN concentrations. TALEN amounts reflect the total TALEN plasmid added to each sample in equal portions. Following electroporation, cells were placed in 6-well plates and allowed to recover for 48 hours. Analysis took place on a Guava EasyCyte 5HT flow cytometer (see materials and methods section). Correction efficiency (%) was determined by the number of viable eGFP positive cells divided by the total number of viable cells in the population. Each treatment was performed in triplicate and error bars represent standard error.

Supplemental Figure S2

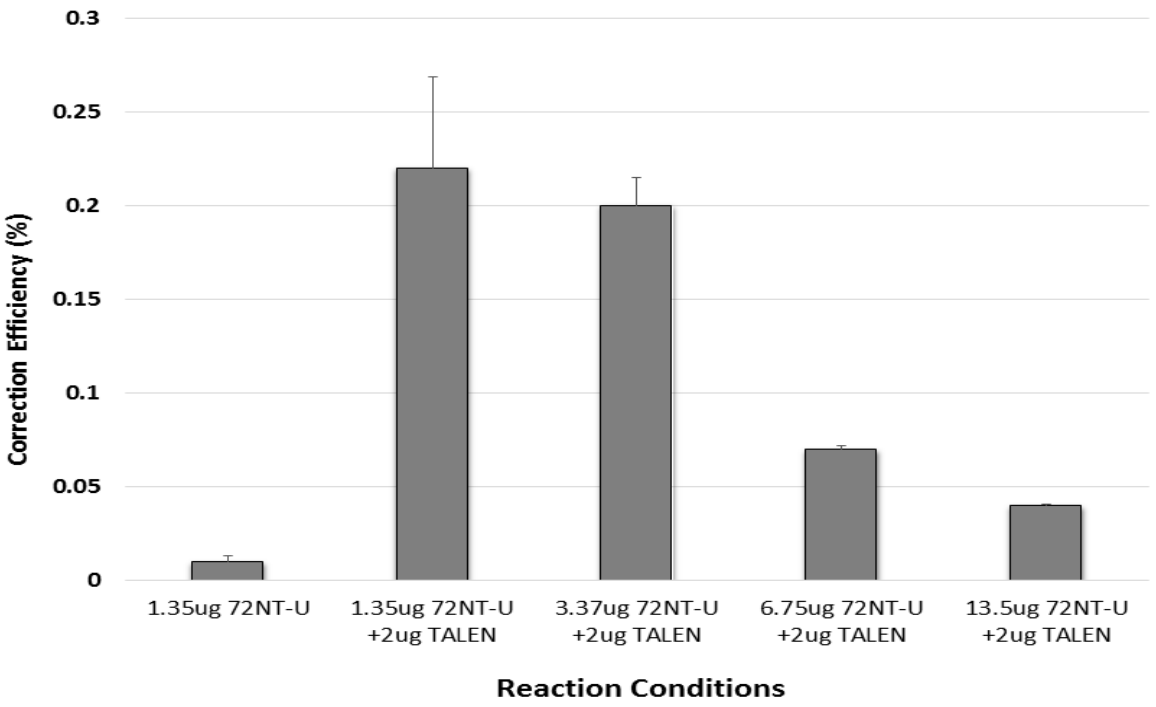

Supplemental Figure S2: Unprotected ssODN and TALENs also support gene editing. Unsynchronized HCT116-19 cells were harvested and electroporated at a concentration of 5e5 cells/100ul with 2ug TALEN (1ug L848-19 and 1ug R898-19) and the above indicated amount of single stranded, unmodified oligonucleotide 72NT-U. Following electroporation, cells were placed in 6-well plates and allowed to recover for 48 hours. Analysis took place on a Guava EasyCyte 5HT flow cytometer (see materials and methods section). Correction efficiency (%) was determined by the number of viable eGFP positive cells divided by the total number of viable cells in the population. Each treatment was performed in triplicate and error bars represent standard error
